# PIF4 Promotes Expression of *HSFA2* to Enhance Basal Thermotolerance in *Arabidopsis*

**DOI:** 10.3390/ijms23116017

**Published:** 2022-05-27

**Authors:** Jiaheng Yang, Xiao Qu, Li Ji, Guanhui Li, Chen Wang, Changyu Wang, Yan Zhang, Lanjie Zheng, Wanchen Li, Xu Zheng

**Affiliations:** 1 State Key Laboratory of Wheat and Maize Crop Science, Center for Crop Genome Engineering, Longzi Lake Campus, College of Agronomy, Henan Agricultural University, Zhengzhou 450046, China; hnndyjh@163.com (J.Y.); 18839770781@163.com (X.Q.); 18482109281@163.com (L.J.); liguanhui2175@163.com (G.L.); wcgfly@163.com (C.W.); 15688179707@163.com (C.W.); yan000zhang@163.com (Y.Z.); zhenglanjie@henau.edu.cn (L.Z.); 2 Key Laboratory of Biology and Genetic Improvement of Maize in Southwest Region, Ministry of Agriculture, Maize Research Institute, Sichuan Agricultural University, Chengdu 611130, China

**Keywords:** heat stress, PIF4, thermotolerance, HSFA2, wheat

## Abstract

Heat stress (HS) seriously restricts the growth and development of plants. When plants are exposed to extreme high temperature, the heat stress response (HSR) is activated to enable plants to survive. Sessile plants have evolved multiple strategies to sense and cope with HS. Previous studies have established that PHYTOCHROME INTERACTING FACTOR 4 (PIF4) acts as a key component in thermomorphogenesis; however, whether PIF4 regulates plant thermotolerance and the molecular mechanism linking this light transcriptional factor and HSR remain unclear. Here, we show that the overexpression of *PIF4* indeed provides plants with a stronger basal thermotolerance and greatly improves the survival ability of *Arabidopsis* under severe HS. Via phylogenetic analysis, we identified two sets (six) of PIF4 homologs in wheat, and the expression patterns of the PIF4 homologs were conservatively induced by heat treatment in both wheat and *Arabidopsis*. Furthermore, the PIF4 protein was accumulated under heat stress and had an identical expression level. Additionally, we found that the core regulator of HSR, HEAT SHOCK TRANSCRIPTION FACTOR A2 (HSFA2), was highly responsive to light and heat. Followed by promoter analysis and ChIP-qPCR, we further found that PIF4 can bind directly to the G-box motifs of the *HSFA2* promoter. Via effector–reporter assays, we found that PIF4 binding could activate *HSFA2* gene expression, thereby resulting in the activation of other HS-inducible genes, such as heat shock proteins. Finally, the overexpression of *PIF4* led to a stronger basal thermotolerance under non-heat-treatment conditions, thereby resulting in an enhanced tolerance to severe heat stress. Taken together, our findings propose that PIF4 is linked to heat stress signaling by directly binding to the *HSFA2* promoter and triggering the HSR at normal temperature conditions to promote the basal thermotolerance. These functions of PIF4 provide a candidate direction for breeding heat-resistant crop cultivars.

## 1. Introduction

As sessile organisms, plants have evolved multiple strategies to sense and cope with many forms of abiotic stress [1,2]. Due to the rising atmospheric CO_2_ concentration [3], high temperatures have been one of the major forces of the suppression of crop yields [4].

In general, the response of plants to temperature can be divided into two forms: acclimation and the tolerance response [5,6]. The definitions of warm temperatures and heat stress (HS) depend on the natural living conditions of specific species [5]. The optimum temperature for *Arabidopsis* is 20–22 °C, with 27–29 °C being considered to be a high ambient temperature [6]. Sessile plants can change their architecture in response to high ambient temperatures (27–29 °C) through elongated hypocotyls and petioles, thinned leaves, and hyponastic growth and early flowering, and these developmental processes have been collectively named thermomorphogenesis [7,8,9]. Temperatures above 30 °C are considered to be HS conditions, and temperatures above 36 °C are considered to be severe HS conditions [10]. HS negatively impacts diverse aspects of plant growth and development [1,2,11,12]. For example, HS-induced protein misfolding results in protein denaturation, plasma membrane destabilization, and reactive oxygen species (ROS) accumulation, causing plant cell death [13,14,15]. When plants suffer from heat stress, the heat stress response (HSR) is induced via H_2_O_2_-induced mitogen-activated protein kinases (MAPKs) and Ca^2+^-dependent calmodulin (CaM3) [16,17,18,19,20,21]. Heat shock transcription factors (HSFs) are the master regulators of the heat stress response and are conserved among eukaryotes [22]. In *Arabidopsis*, there are 21 HSFs that can be grouped into three classes: HSFA, HSFB, and HSFC [23]. When plant cells sense heat stress, the HSFA1s act as master activation regulators of various *HSR* genes that include not only *heat shock proteins* (*HSPs*) but also TFs, such as *HSFA2*, *HSFA3*, *HSFA7a*, *HSFA7b*, *HSFB1*, *HSFB2a*, *HSFB2b*, and dehydration-responsive element binding 2A (*DREB2A*) [11,13,22,24,25,26].

Previous studies have established that *HSFAs* and *DREB2A* are positive regulators of HSR and that they can enhance and maintain the expression of *HSR* genes and amplify the heat stress response to enhance thermotolerance in plants [27,28,29,30,31]. HSFA1 directly targets *DREB2A*, further activating *HSFA3* expression to maintain the heat stress response for longer periods of time [29,30]. Increasing data indicate that *HSFA2* is the most highly heat-induced *HSF* in *Arabidopsis* and that the acquired thermotolerance is completely abolished in *hsfa2* knockout mutants [11,28,32]. *HSFA2* is also related to HS memory in plants [28]. When plants are exposed to a mild primary HS, H3K4me2 and H3K4me3 are remarkably enriched at the loci of HS memory genes (*APX2*, *HSP18.2,* and *HSP22*), leading to a higher and longer expression of the HS memory genes, which are key regulators of acquired thermotolerance [32,33,34]. After mild primary HS, HSFA3 binds to HSFA2 and then forms heteromeric complexes that are essential for the accumulation of H3K4me2 and H3K4me3, thereby resulting in enhanced HS memory [21]. HSFA2 also establishes a heritable feedback loop with the H3K27me3 demethylase RELATIVE OF EARLY FLOWERING 6 (REF6), ensuring transgenerational thermomemory in *Arabidopsis* [35].

HSPs function as molecular chaperones that are responsible for assisting stress-induced misfolded protein folding to maintain cell homeostasis [11,36]. Under normal temperature conditions, HSP70 and HSP90 negatively regulate the nuclear localizations and activities of HSFA1s, whereas heat stress induces the accumulation of unfolded proteins that interact competitively with HSP70/HSP90, thereby causing HSFA1s to be released from the HSP70/HSP90 complex and become active [22,37,38]. Plants have evolved multiple molecular mechanisms to activate the heat stress response; however, there is little information on the linking of the HSR and light signaling transcription factors, and the underlying regulatory mechanism remains unclear.

Previous studies have established that PHYTOCHROME INTERACTING FACTOR 4 (PIF4), a bHLH transcription factor, acts as a key component in thermomorphogenesis [39]. The transcription of PIF4 is induced by high ambient temperatures [40], thereby enhancing the accumulation of PIF4 protein. The increased PIF4 protein induces hypocotyl growth by activating the expression of auxin biosynthetic genes such as *YUCCA8 (YUC8)*, *CYP79B2,* and *TAA1*; auxin signaling genes such as *IAA19* and *IAA29*; growth-promoting genes such as *ATHB2* and *LNGs*; and brassinosteroid biosynthetic genes such as *BES1* and *BZR1* [41]. The evening complex (EC), which consists of *ELF3*, *ELF4*, and *LUX*, mediates the warm-temperature activation of *PIF4* mRNA expression [42]. EC represses *PIF4* and *PIF5* expression by directly binding to their promoters [43,44]. Very recently, PIF4 was shown to play a critical role in mediating *Arabidopsis* leaf senescence induced by heat stress (42 °C) [45].

Nevertheless, previous studies have established that PIF4 is a core hub of thermomorphogenesis when plants are exposed to high ambient temperatures [40,41,46,47,48,49,50]. However, whether PIF4 also regulates HSR when plants are subjected to severe HS and the mechanistic details underlying this proceeding remains unclear. Here, we demonstrate that the high-level overexpression of *PIF4* significantly enhances the survival rate of *Arabidopsis* seedlings after severe HS. PIF4 binds directly to the *HSFA2* promoter and positively regulates its expression, leading to a stronger capacity to withstand HS and the enhanced basal thermotolerance of plants. Additionally, the heat responses of PIFs are conserved in wheat; this may provide a candidate direction for breeding heat-resistant crop cultivars to ensure food security.

## 2. Results

### 2.1. Phylogenetic Analysis of PIFs in Dicot and Monocot Plants

To investigate whether the PIF proteins are conserved across plants and their application values, we performed phylogenetic analysis on the PIFs in dicot (*Arabidopsis thaliana*, *Solanum lycopersicum*, and *Glycine max*) and monocot plants (*Triticum aestivum*, *Zea mays*, and the *Oryza sativa Japonica* *Group*). The proteins containing at least one motif (APA or APB) and a bHLH domain were selected for phylogenetic tree construction.

**Figure 1:**
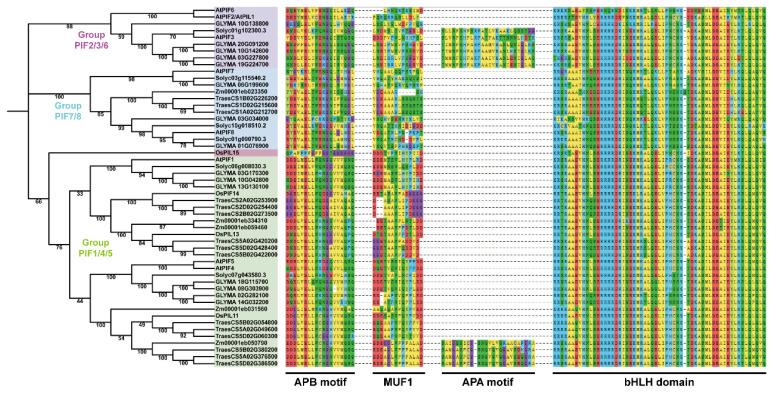
A neighbor-joining (NJ) phylogenetic tree of PIFs in *Arabidopsis thaliana*, *Solanum lycopersicum*, *Glycine max*, *Triticum aestivum*, *Zea mays,* and the *Oryza sativa Japonica Group*. The full-length protein sequences were used for the construction of a phylogenetic tree using MEGAX. The tree showed four major groups, which are indicated with different backgrounds. Multiple sequence alignment and motif analysis were performed using MEGAX and MEME.

The phylogenetic tree is presented in Figure 1. The tree shows that all of the PIF genes in the plants could be classified into four groups: group PIF2/3/6, group PIF7/8, group PIF1/4/5, and OsPIL15, which is consistent with previous studies [51,52]. The protein domains were highly conserved in dicot and monocot plants, indicating the evolutionary and functional conservation among PIF gene family members.

### 2.2. PIFs Positively Regulated Thermotolerance under Severe HS

The PIFs were found to be responsible for thermomorphogenesis, regardless of whether or not it contributed to thermotolerance. To investigate whether phytochrome interacting factors (PIFs) regulate thermotolerance in plants, we first performed thermotolerance phenotype assays to test the survival rate of WT and *pifq* (*pif1/3/4/5* quadruple mutant) after severe HS.

**Figure 2:**
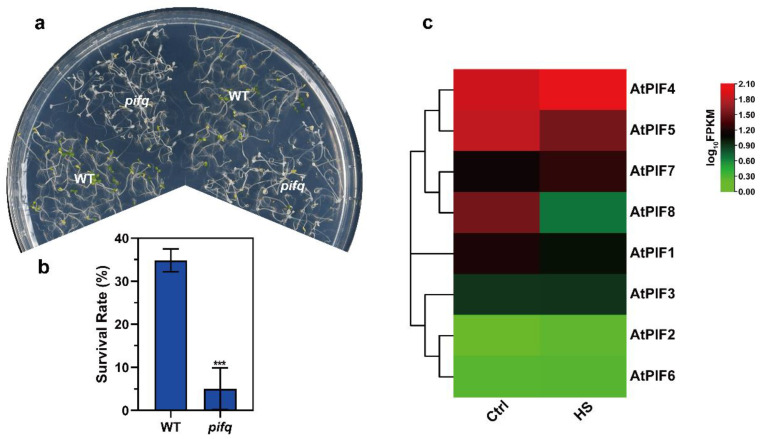
The pifq (pif1/3/4/5) mutant exhibits reduced thermotolerance. (**a**) Thermotolerance phenotypes of 4-day-old pifq seedlings grown in darkness were treated at 45 °C for 1 h and were allowed to recover at 22 °C for 3 d under long day conditions (16 h light/8 h darkness). (**b**) Quantification of the survival rates as indicated in (**a**). Values are the means ± SD of three independent biological replicates. Statistical differences calculated using Student’s *t*-test are shown. Asterisks indicate the significant differences (*** *p* < 0.001). (**c**) Heat maps of the expression of the PIF genes under heat stress. The FPKM values of each gene are normalized by log10.

As the PIFs were accumulated under dark conditions, we performed the thermotolerance phenotype assays using seedlings that had been grown in darkness for 4 days. Then, we treated the seedlings at 45 °C for 1 h and allowed them to recover at 22 °C for 3 days under long day conditions (16 h light/8 h darkness). Compared to WT, the *pifq* seedlings exhibited remarkably weaker thermotolerance (Figure 2a). Consistently, the survival rate data revealed that severe HS-induced plant death was enhanced in the *pifq* mutant (5.05%) (Figure 2b). These results suggest that PIFs positively regulate thermotolerance under severe HS in plants.

Previous studies showed that there were eight members in the *Arabidopsis PIF* family: *PIF1*, *PIF2*, *PIF3*, *PIF4*, *PIF5*, *PIF6*, *PIF7*, and *PIF8* [53,54]. To determine which PIF gene was induced by heat stress, we next checked the PIF expression pattern on 7-day-old WT seedlings under normal temperature and HS conditions (37 °C for 1 h). The heat map of the *PIFs* under normal temperature and HS conditions revealed that *PIF4* was up-regulated under HS, but that the other *PIFs* were not (Figure 2c). Taken together, these observations led us to investigate the roles of *PIF4* under severe HS further.

### 2.3. Spatio-Temporal Expression Analysis of PIFs in Arabidopsis and Triticum

**Figure 3:**
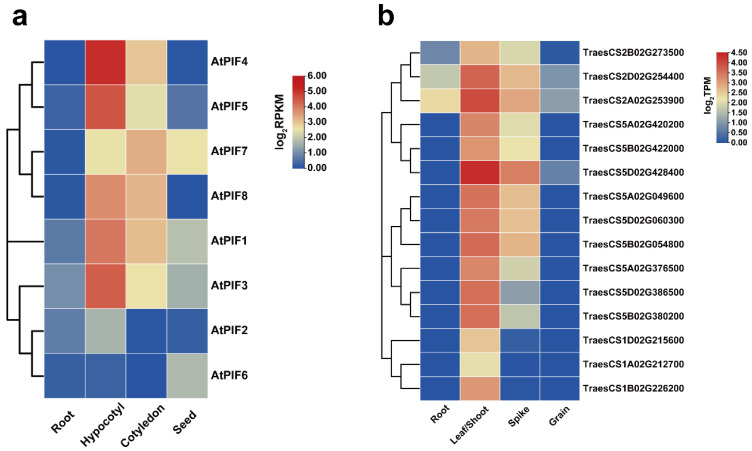
Expression analysis of *PIFs* in different *Arabidopsis* and *Triticum* tissues. (**a**) Hierarchical clustering of expression profiles of *AtPIF* genes in different tissues. The RPKM values of each gene are normalized by log2. (**b**) Hierarchical clustering of the expression profiles of *TaPIF* genes in different tissues. The TPM values of each gene are normalized by log2.

The transcriptome data were downloaded from public databases, and the expression patterns of the 8 *AtPIFs* and 15 *TaPIFs* in different tissues (root, hypocotyl/shoot, cotyledon/leaves, spike, and seed) were characterized (Figure 3). The result revealed that the *AtPIFs* and *TaPIFs* exhibited similar expression patterns in different tissues. The PIFs were described as the master negative regulator of photomorphogenesis in previous studies [53,54]. We found that the expression levels of *AtPIFs* and *TaPIFs* in the hypocotyl and cotyledon or in leaves and shoots were remarkably higher than they were in roots and seeds or in grains (Figure 3). This indicated that the function of PIFs on photomorphogenesis is conserved among *Arabidopsis* and *Triticum*.

### 2.4. TaPIF4 Exhibited Similar Transcription Patterns in Response to Heat Stress

The phylogenetic tree showed that the *AtPIF4* homolog genes in wheat were probably *TraesCS5A02G049600*, *TraesCS5D02G060300, TraesCS5B02G054800* and *TraesCS5A02G376500*, *TraesCS5D02G386500,* and *TraesCS5B02G380200*, which were in the clade and are closely associated with *AtPIF4* (Figure 1).

**Figure 4:**
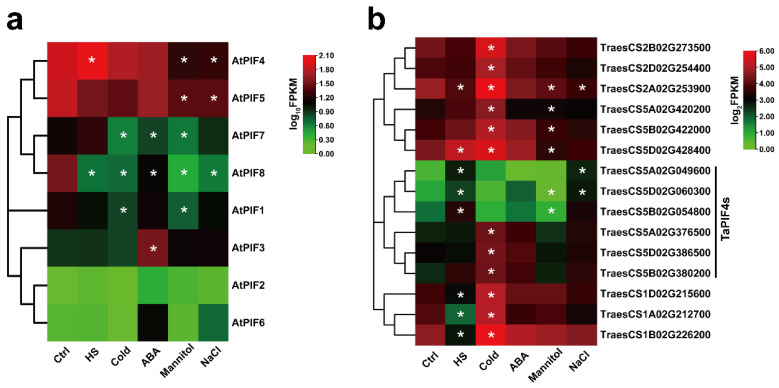
Expression analysis of the PIFs of Arabidopsis and Triticum under different abiotic stress treatments. (**a**) Hierarchical clustering of the expression profiles of AtPIF genes under different abiotic stress treatments. The FPKM values of each gene are normalized by log10. (**b**) Hierarchical clustering of the expression profiles of TaPIF genes under different abiotic stress treatments. The FPKM values of each gene are normalized by log2. Statistical differences calculated by DESeq2 are shown. Asterisks indicate the significant difference (* *p* < 0.05).

We then performed RNA-seq on 7-day-old Col-0 seedlings and 2-week-old AK58 wheat cultivar seedlings treated with different abiotic stresses. The data showed that the expression of *TraesCS5A02G049600*, *TraesCS5D02G060300*, and *TraesCS5B02G054800* were significantly up-regulated under HS conditions (Figure 4b), consistent with *AtPIF4* (Figure 4a). Interestingly, *AtPIF4* was only up-regulated in response to HS but down-regulated in response to other types of abiotic stress, such as Mannitol and NaCl (Figure 4a). *TraesCS5A02G049600* and *TraesCS5D02G060300* were up-regulated under NaCl stress. Moreover, *TraesCS5A02G376500*, *TraesCS5D02G386500,* and *TraesCS5B02G380200* were induced significantly by the cold treatment.

These observations suggest that the *AtPIF4* homolog genes in wheat exhibited similar transcription patterns in response to heat stress but diverse responses to other abiotic stresses, such as NaCl and cold.

### 2.5. PIF4 Was a Key Factor for Plant Thermotolerance under Severe HS

Previous studies have well established that PIF4 is a core hub of thermomorphogenesis and photomorphogenesis in plants [6,55]. In darkness, PIF4 promotes skotomorphogenic development, thereby leading to rapid hypocotyl elongation [56]. The photomorphogenesis phenotype assay showed that pifq and the PIF4 loss-of-function mutant (pif4-1) exhibited a shorter hypocotyl compared to the WT under red light; in contrast, the overexpressed PIF4 (PIF4-OE) exhibited a longer hypocotyl (Figure S1), consistent with previous studies [53,54,57].

**Figure 5:**
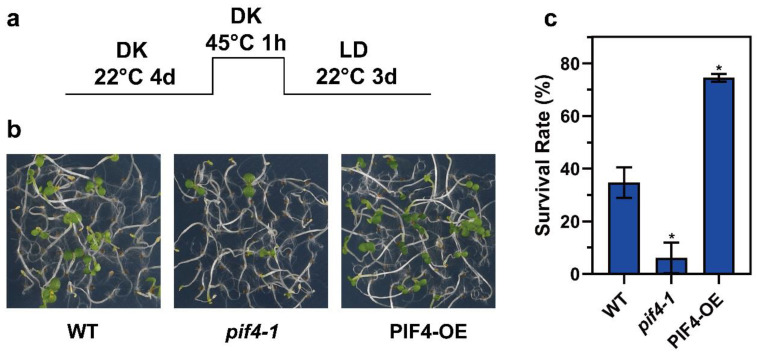
*PIF4* overexpression enhances the thermotolerance of *Arabidopsis*. (**a**) Col-0, *pif4-1,* and PIF4-OE seedlings were grown in darkness for 4 d and then treated at 45 °C for 1 h and recovered at 22 °C for 3 d under long day conditions (16 h light/8 h darkness). (**b**) The thermotolerance phenotypes of Col-0, *pif4-1,* and PIF4-OE were shown after HS treatment, as indicated in (**a**). (**c**) Quantification of the survival rates indicated in (**a**). Values are the means ± SD of three independent biological replicates. Statistical differences calculated by Student’s *t*-test are shown. Asterisks indicate the significant differences (* *p* < 0.05).

We wondered whether PIF4 was involved in thermotolerance regulation. Next, we performed the thermotolerance phenotype assay using WT, pif4-1, and PIF4-OE. Under severe HS conditions (Figure 5a), the pif4-1 plants exhibited weaker acquired thermotolerance compared to WT, and on the contrary, the PIF4-OE showed stronger thermotolerance (Figure 5b), which was evident in the lower survival rate of pif4-1 (6.12%) and the higher survival rate of PIF4-OE (74.49%) compared to WT (34.69%) (Figure 5c). In summary, these results demonstrate that PIF4 is not only a core hub of thermomorphogenesis but also a key factor for plant thermotolerance under severe HS conditions.

### 2.6. Heat Stress Induced the Accumulation of PIF4

**Figure 6:**
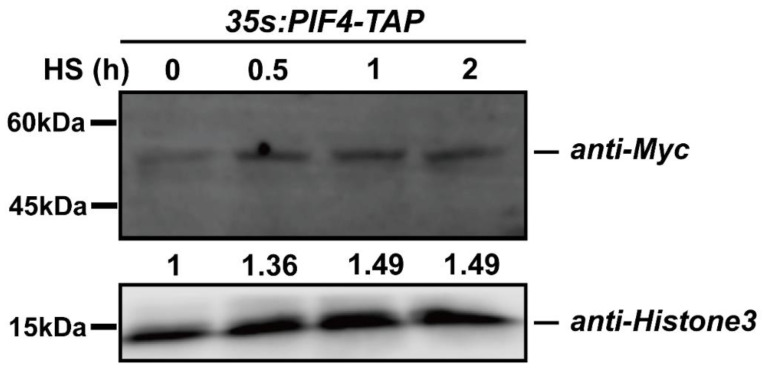
The protein accumulation of PIF4 under heat stress conditions. Detection of the PIF4 protein levels in 4-day-old *35S:PIF4-TAP/WT* seedlings grown in darkness and in the seedlings transferred to 45 °C for 0.5, 1, and 2 h. The proteins were detected by immunoblot analysis using an anti-Myc antibody.

We found that PIF4 expression was induced by heat stress (Figure 2c), and we wondered whether the post-transcription of PIF4 was regulated by the severe HS. To rule out the expression effect, we chose constitutive promoter-driven PIF4. Additionally, the qPCR data showed that the expression levels of PIF4 were not induced by heat shock treatment in the *35S:PIF4-TAP/WT* seedlings and that even the expression value of PIF4 after HS for 2 h was down-regulated (Figure S2). Next, the immunoblot assay was performed to detect PIF4 protein accumulation using *35S:PIF4-TAP/WT* seedlings under 45 °C for 0.5, 1, and 2 h. The TAP-tag consisted of His, Myc, and Flag, so the *35S:PIF4-TAP/WT* seedlings were first detected by using the anti-Myc primary antibody (Figure S3). After the severe HS treatment, the total proteins were extracted with lysis buffer and then immunoblotted with an anti-Myc primary antibody and a horseradish peroxidase-conjugated anti-mouse IgG secondary antibody. Remarkably, the abundance of PIF4 proteins increased with the HS treating time and remained unchanged for up to 2 h, indicating that HS induces PIF4 accumulation (Figure 6). Taken together, severe heat stress induces the post-transcriptional accumulation of PIF4, and this might be critical for the plant response to severe heat stress.

### 2.7. Overexpression of PIF4 Enhanced the Basal Expression of HS-Inducible Genes

**Figure 7:**
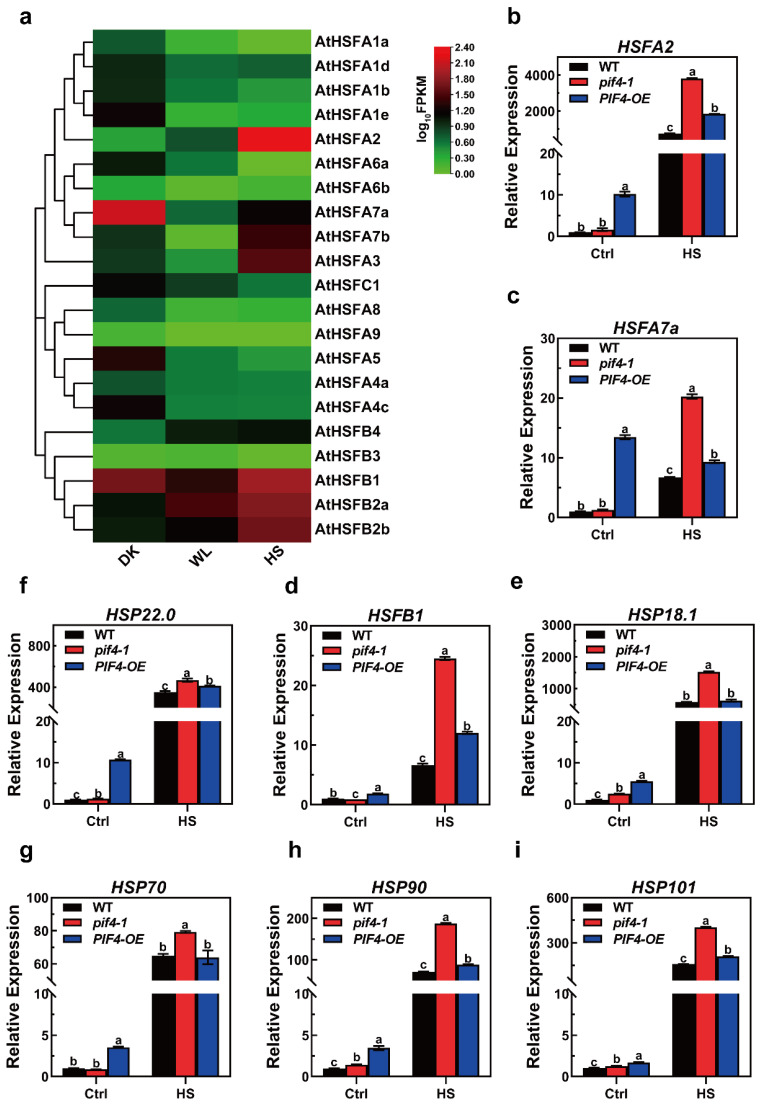
The expression patterns of heat stress (HS)-inducible genes under severe heat stress conditions. (**a**) Heat maps of the expression of the AtHSF genes in 7-day-old WT seedlings under DK, WL (16 h light/8 h darkness), and HS (16 h light/8 h darkness, 37 °C for 1 h). The FPKM values of each gene are normalized by log10. (**b**–**i**) Relative expression levels of HS-inducible genes in overexpressed WT, pif4-1 mutant, and PIF4-OE lines under control (Ctrl) and HS conditions (45 °C for 1 h). Gene expression levels are expressed relative to the value in non-heat-treated Col-0 seedlings. Error bars indicate the mean ± SD. Actin2 was used as a reference gene. Statistical differences were calculated by one-way ANOVA. Different letters above each bar indicate statistically significant differences as determined by Tukey’s multiple testing methods (*p* < 0.05).

Heat shock factors (HSFs) are known as key regulators in the response to heat stress. Here, we found that light signaling factor PIF4 was responsible for the plant heat stress response. Additionally, there were 21 HSFs in Arabidopsis, and we wondered if there could be an HSF that could be regulated by both light and heat signaling. According to this hypothesis, an HSF could work as a linker to connect light to thermotolerance. To investigate which HSF responds to both light and heat, we checked the expression profiles of all 21 HSFs in darkness and white light (WL, 16 h light/8 h darkness) and under high temperatures (16 h light/8 h darkness, 37 °C for 1 h). With the results, we had a heat map to indicate the expression patterns, and there were four HSFs that could be induced by light and heat, HSFA2, HSFB2a/b, and HSFB4. Unsurprisingly, HSFA2 was induced the most in heat stimuli, as it was previously determined to be the main regulator of acquired thermotolerance (Figure 7a). For the HSFBs, although they were induced by heat, they were not involved in plant thermotolerance regulation [58,59]. As such, HSFA2 might work as a linker to connect the PIF4 to thermotolerance regulation.

The opposite thermotolerance phenotype of the pif4-1 mutants and PIF4-OE prompted us to explore the differences in the transcriptional levels of the heat stress (HS)-inducible gene associated with basal and acquired thermotolerance between WT, pif4-1, and PIF4-OE.

Notably, we also observed that compared to WT and pif4-1, the transcriptional levels of all of the HS-inducible genes were significantly higher in PIF4-OE under non-heat conditions (Figure 7b–i). Together, these data indicate that the basal thermotolerance of PIF4-OE is stronger than that WT and pif4-1 and demonstrate that these are probably the critical reasons why PIF4-OE has a higher survival rate than WT under severe HS.

Surprisingly, the qRT-PCR results showed that the expression levels of HSFA2, HSFA7a, HSFB1, HSP22.0, HSP90, and HSP101 were significantly up-regulated in both pif4-1 and PIF4-OE compared to those in WT plants under severe HS. Moreover, the transcriptional levels of the HS-inducible genes in pif4-1 were even higher than PIF4-OE under severe HS (Figure 7). These results showed that PIF4 regulated the expression of HSR genes under severe HS. Nevertheless, the weaker thermotolerance of pif4-1 indicates that PIF4 not only regulates the transcription of HSR genes but that it also might impact the post-transcriptional levels or modification of HSR proteins. The presence or absence of PIF4 might affect the function of these HSR genes under severe HS. 

### 2.8. PIF4 Directly Bound to the Promoter of HSFA2 and Promoted Its Gene Expression

**Figure 8:**
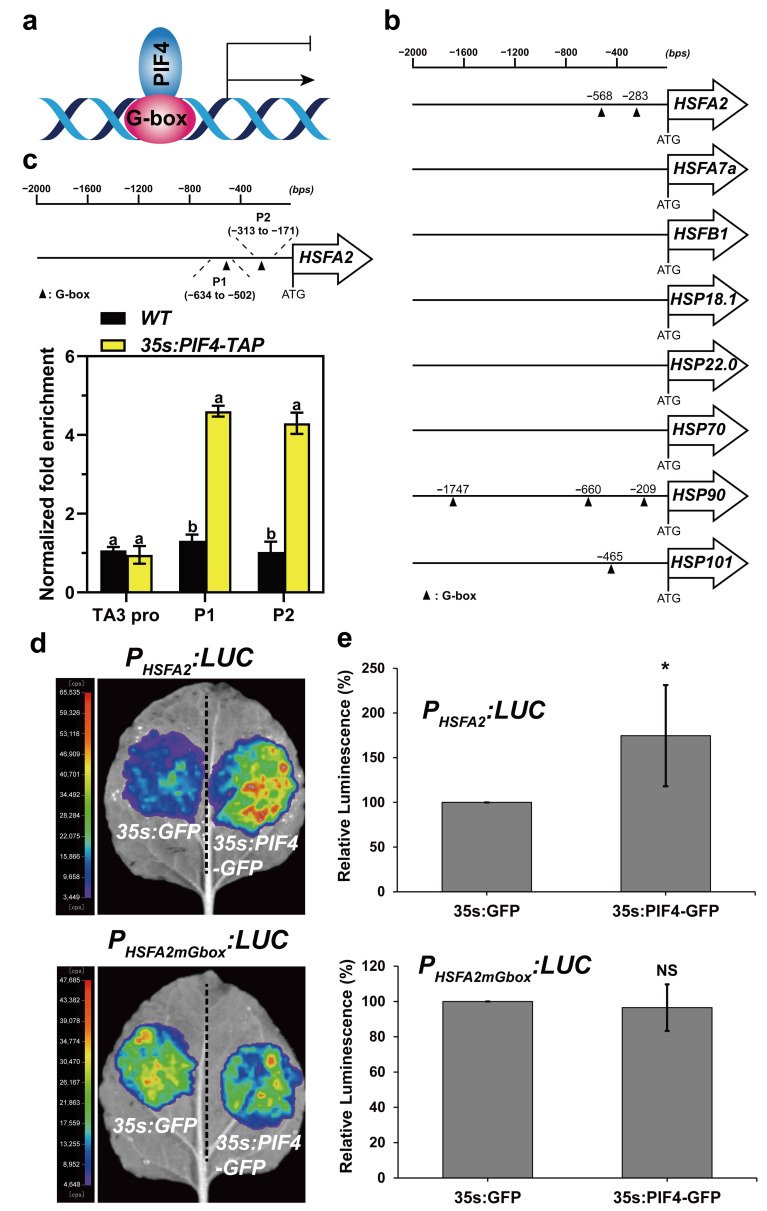
PIF4 directly binds to the G-box motifs in the HSFA2 promoter. (**a**) PIF4 directly binds to the G-box motif of gene promoters to activate or repress their expression. (**b**) Schematic representation of the HSR gene promoters with the location of the G-box motif. The triangle indicates the G-box motif. (**c**) Schematic representation of the HSFA2 promoter with the location of two G-box motifs. P1 and P2 represent the respective primer positions used for ChIP-qPCR. ChIP-qPCR analysis of PIF4 binding to the HSFA2 promoter. ChIP assays were performed on 7-day-old WT and *35s:PIF4-TAP* seedlings grown at 22 °C in darkness. The protein–DNA complexes were immunoprecipitated using anti-Myc beads. ChIP DNA was quantified by qRT-PCR with primers specific to the G-box motifs (P1 and P2) and TA3 promoter (negative control). The ChIP-qPCR values of these regions were normalized to TUB2. The enrichment level in WT was set to 1. Values are the means ± SD of four technical replicates. Statistical differences were calculated by one-way ANOVA. Different letters above each bar indicate statistically significant differences as determined by Tukey’s multiple testing methods (*p* < 0.05). (**d**) Activation of the HSFA2 promoter by PIF4 in Nicotiana benthamiana leaves. A reporter vector P_HSFA2_:LUC containing the HSFA2 promoter (1500 bp upstream of the start codon) driving LUC. P_HSFA2mGbox_:LUC represents the G-box mutation of P_HSFA2_:LUC, and 35s:GFP (negative control) and 35s:PIF4 were used as effector constructs. (**e**) The quantification of the luciferase activity for the samples shown in (**b**,**d**). All the 35s:GFP controls were set at 100%. The ratio represents the relative luminescence intensity of the 35s:PIF4 effector compared to the 35s:GFP effector in the same reporter. Data are means of relative luminescence ratios and error bars represent SD of relative luminescence ratios for at least four independent biological replicates. Asterisks indicate statistically significant differences (* *p* < 0.05, NS, no significance).

It was previously demonstrated that PIF4 directly activates or represses the expression of downstream genes via binding to G-box (CACGTG) and/or PBE-box (CACATG) motifs in their promoters (Figure 8a) [60,61,62]. Next, we analyzed the promoter structures of the above HS-inducible genes. The results showed that the promoter regions of HSFA2, HSP90, and HSP101 harbored 1 to 3 G-box motifs (Figure 8b). This observation indicated that PIF4 might bind directly to the loci of HSFA2, HSP90, and HSP101 to regulate their transcription. HSFA2 was the most highly heat-induced HSF, representing the master regulator, and played a critical role in the acquired thermotolerance of Arabidopsis [28,31]. Moreover, the expression of HSFA2 was highly responsive to light and heat (Figure 7a).

We next performed ChIP-qPCR assays using 7-day-old WT and *35s:PIF4-TAP* seedlings grown at 22 °C in darkness to investigate whether PIF4 could directly regulate the expression of HSFA2 via binding to the G-box motifs under non-heat conditions. The specific primers were designed to detect the enrichment of the HSFA2 promoter of the “P1” and “P2” regions harboring G-box motifs (Figure 8c), and the enrichment in the TA3 promoter was used as a negative control [63]. The results showed that the “P1” and “P2” regions of the HSFA2 promoter were remarkably enriched in the ChIP DNA samples from *35s:PIF4-TAP* seedlings but not in the WT seedlings compared to the TA3 promoter region (Figure 8c).

To further investigate whether the binding of PIF4 in the loci of the HSFA2 promoter is responsible for regulating its expression, we performed an effector–reporter assay in Nicotiana benthamiana leaves to confirm this hypothesis. As expected, 35s:PIF4-GFP but not 35s:GFP elevated the LUC activity of P_HSFA2_:LUC remarkably (Figure 8d,e). Interestingly, the promotion of PIF4 to the HSFA2 promoter was abolished in P_HSFA2mGbox_:LUC, which is the G-box mutant of P_HSFA2_:LUC reporters, confirming that the positive role of PIF4 on HSFA2-driven transcriptional activation is reliant on G-box motifs (Figure 8d,e). Collectively, these results suggest that PIF4 directly binds to the G-box motifs in the HSFA2 promoter to promote its expression under non-heat conditions, leading to enhanced basal thermotolerance in PIF4-OE.

## 3. Discussion

In recent years, global warming has caused frequent occurrences of extreme weather, which have had a detrimental impact on plant growth and development throughout the whole plant life cycle [64]. As sessile organisms, plants cannot migrate to avoid extreme temperatures; therefore, multiple strategies have evolved to sense and cope with heat stress in plants [6,8,21]. In general, extreme temperatures display two forms: high ambient temperatures (27~29 °C) and heat stress (≥30 °C) [5,6]. It is worth noting that temperatures above 36 °C are considered to be severe HS for Arabidopsis [6]. Phytochrome interacting factors (PIFs) have been reported to play a critical role in seedling development, hypocotyl elongation, and thermomorphogenesis [55,60]. In addition to this, our data showed that PIF4 can be involved in thermotolerance regulation and how it functions.

### 3.1. The Expression Patterns of PIF4 in Response to Heat Stress Were Highly Conserved in Arabidopsis and Triticum

Previous studies have demonstrated that PIFs contain two highly conserved motifs: the active PHYB binding (APB) and active PHYA binding (APA) motifs, which are necessary for interaction with PHYB and PHYA [53,65,66]. MUF1 is reported to play a critical role in the transcriptional activation of PIFs [67]. Furthermore, the basic/helix–loop–helix domain is highly conserved in bHLH transcription factors [68]. We next performed a BLASTP search using Ensemble Plants (http://plants.ensembl.org/index.html, accessed on 11 April 2022) and chose the sequences with an e-value less than 1 × 10^−10^. The MUF1 and bHLH domain sequences were defined by MEME and were consistent with previous studies [51,68]. The phylogenetic tree showed that all of the PIF genes of plants could be classified into three groups: PIF2/3/6, PIF7/8, and PIF1/4/5, consistent with previous studies [51,52]. The highly conserved protein domains in the dicot and monocot plants indicated the evolutionary and functional conservation among PIF gene family members (Figure 1). Meanwhile, the homology analysis of the PIFs in Arabidopsis and Triticum showed that there are two clusters of AtPIF4 homologs in wheat (Figure 1). We found that the expression levels of the AtPIFs and TaPIFs in the hypocotyl and cotyledon or in leaves and shoots were remarkably higher than in roots and seeds or grains (Figure 3). This indicated that the function of PIFs in photomorphogenesis is conserved among Arabidopsis and Triticum.

In this study, the seedlings lacking PIF genes (pifq mutants) caused a drastic reduction in the survival rate under severe HS, and PIF4 expression was induced by heat stress (Figure 2). Furthermore, AtPIF4 and the predicted TaPIF4s (TraesCS5A02G049600, TraesCS5D02G060300, TraesCS5B02G054800, TraesCS5A02G376500, and TraesCS5B02G380200) exhibited similar transcription patterns in response to heat stress but diverse responses to other types of abiotic stress, such as NaCl, cold, and ABA (Figure 4), indicating that the function of PIF4s was conserved in response to heat stress.

### 3.2. PIF4 Was Essential for Plants in Response to the Severe Heat Stress (45 °C)

Previous studies have established that PIF4 acts as a key component in thermomorphogenesis, which changes the morphology architecture of plants in response to moderately high ambient temperature [40,50,69]. In more detail, PIF4 can activate the expression of auxin biosynthetic genes such as YUCCA8 (YUC8), CYP79B2, and TAA1; auxin signaling genes such as IAA19 and IAA29; growth-promoting genes such as ATHB2 and LNGs; and brassinosteroid biosynthetic genes such as BES1 and BZR1 in response to high ambient temperatures [39,42,46,48,49,70,71]. Recent studies have also shown that PIF4 and PIF5 mediate heat-stress-induced leaf senescence [45]. Nevertheless, the molecular mechanisms of PIF4 underlying severe heat stress (45 °C) remain unclear.

To clarify the role of PIF4 in the heat stress response of plants, we investigated the thermotolerance phenotype of pif4-1 and PIF4-OE under severe HS. It is known that PIF proteins accumulate in darkness when plants are exposed to light and when the phytochromes interact with PIFs, leading to their rapid turnover via ubiquitination and phosphorylation and the mediation of the PIF degradation via the 26S proteasome pathway [65,72,73,74,75,76]. As such, we performed thermotolerance phenotype assays using seedlings grown in darkness for 4 days. The seedlings were then treated at 45 °C for 1 h and recovered at 22 °C for 3 days under long day conditions (16 h light/8 h darkness).

Then, we found that the death of the seedlings with induced heat stress was remarkably reduced in PIF4-OE and significantly enhanced in pif4-1 compared to WT (Figure 5a), which was evident in the lower survival rate of pif4-1 (6.12%) and higher survival rate of PIF4-OE (74.49%) compared to WT (34.69%) (Figure 5b). Furthermore, to investigate PIF4 regulation at the post-transcriptional level by severe HS, we detected the protein accumulation of PIF4 under 45 °C for 0.5, 1, and 2 h. Additionally, the PIF4 protein levels increased as the HS treatment time was prolonged (Figure 6). Heat promoted the transformation of PHYB from Pfr to Pr, which might inhibit PIF4 protein degradation [77,78]. The increased PIF4 protein accumulation might play a critical role in the response of plants to severe heat stress.

### 3.3. PIF4 Directly Bound to the Promoter of HSFA2 to Active Its Expression

Recent studies have indicated that HSFA2 and HSFA7a are positive regulators of HSR and that they maintain HSR gene expression, leading to plants having enhanced thermotolerance [25,28]. It was found that HSFA2 and HSFA7a could directly bind to the heat stress elements of HSPs to activate their expression and gain a stronger and longer HSR for the heat acclimation response [25,28]. It is worth noting that HSPs function as molecular chaperones and are involved in the HS-induced unfolded protein response to maintain plant cell homeostasis [11,36]. Moreover, HSFB1 repressed the expression of the heat-stress-induced HSFs but is necessary for the acquired thermotolerance [58].

In the current study, we found that the transcriptional levels of HS-inducible marker genes were significantly higher in PIF4-OE compared to in WT and pif4-1 under non-heat conditions (Figure 7). These results showed that the transcriptional levels of HS-inducible genes were regulated by PIF4. Furthermore, these data also indicated that the higher transcriptional levels of HSR genes might result in the stronger basal thermotolerance of PIF4-OE than WT and pif4-1. Additionally, it was probably the critical reason for the higher survival rate of PIF4-OE than WT under severe HS.

Surprisingly, the qRT-PCR results showed that compared to WT, the transcriptional levels of HSFA2, HSFA7a, HSFB1, HSP22.0, HSP90, and HSP101 were significantly up-regulated in both pif4-1 and PIF4-OE after severe HS treatment, and all of the transcriptional levels of the HS-inducible genes in pif4-1 were higher than PIF4-OE under severe HS (Figure 7). Nevertheless, the higher transcriptional levels of HS-inducible genes in pif4-1 did not rescue its heat-resistant phenotype (Figure 5). This result suggests that PIF4 not only regulates the transcription of HSR genes but that it also might mediate the post-transcriptional regulation of HSR proteins. The presence or absence of PIF4 may determine the positive function of these HSR genes under severe HS, and this field requires further investigation in the future.

Due to PIF4 directly binding to the G-box (CACGTG) and/or PBE-box (CACATG) motifs of downstream genes to activate or repress their expression [60,61,62], we next investigated the possibility of PIF4 binding to the promoters of these HS-inducible genes. The promoter sequence analysis showed that HSFA2, HSP90, and HSP101 harbor one to three G-box motifs in their promoter regions (Figure 8b). HSFA2 is considered to be the most highly heat-induced HSF and plays a critical role in the acquired thermotolerance of Arabidopsis. Next, we performed the ChIP-qPCR assay to confirm this hypothesis. The results showed that the “P1” and “P2” regions of the HSFA2 promoter were remarkably enriched in the ChIP DNA samples from the *35s:PIF4-TAP* seedlings but not in the WT seedlings compared to the TA3 promoter region (Figure 8c), indicating that PIF4 can bind directly to the loci of HSFA2 in vivo. Furthermore, effector–reporter assays showed that the binding of PIF4 to the G-box motifs elevated the transcriptional levels of HSFA2 remarkably (Figure 8d,e).

**Figure 9:**
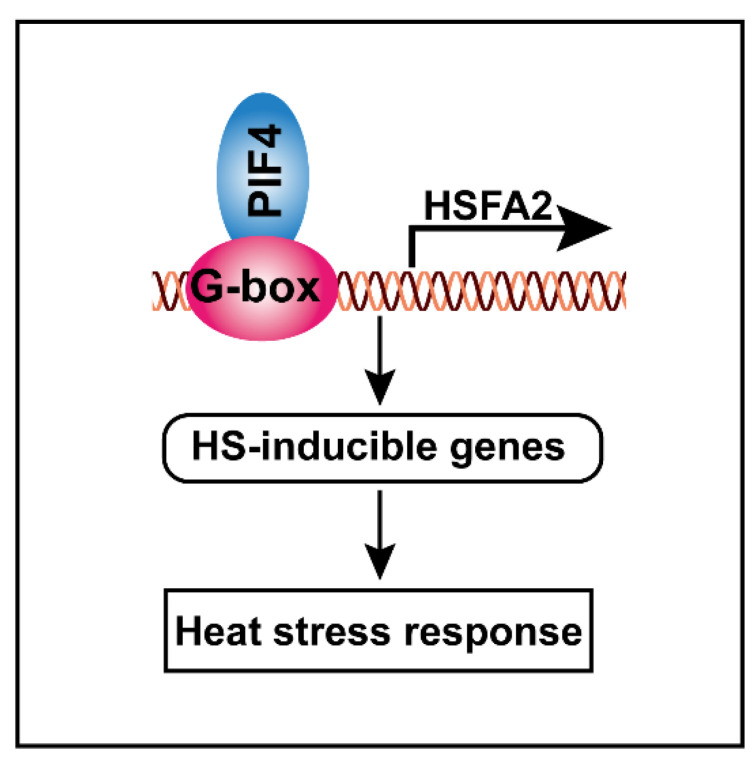
Schematic model showing how PIF4 modulates the heat stress response in plants. PIF4 directly binds to the G-box motifs to promote *HSFA2* gene expression, thereby resulting in the activation of the HS-inducible genes. These leads to a stronger basal thermotolerance under non-heat-treatment conditions, thereby resulting in enhanced tolerance to severe heat stress.

Taking these results together, we proposed a hypothetical model that elucidates the molecular function of PIF4 in response to severe heat stress. PIF4 can directly bind to the G-box motifs of the HSFA2 promoter to activate its gene expression, thereby resulting in the activation of other HS-inducible genes, such as heat shock proteins. These lead to a stronger basal thermotolerance under non-heat-treatment conditions, resulting in higher tolerance to severe heat stress (Figure 9).

The elevated thermotolerance of crops is critical for ensuring food security. Here, we found that the overexpression of PIF4 could provide a stronger basal thermotolerance for plants, greatly improving the survival ability of Arabidopsis under severe HS, and the AtPIF4 homolog genes in wheat exhibited similar transcription patterns in response to heat stress. Our findings establish a molecular mechanism of PIF4 in mediating the heat stress response and provide a candidate direction for breeding heat-resistant wheat cultivars. Although our research yielded a model for PIF4 modulating the heat stress response to cope with severe HS rather than thermomorphogenesis, a number of questions remain unclearly answered. These include why pif4-1 had stronger transcriptional levels of HS-inducible genes but the thermotolerance was still weaker. Other questions include whether the post-transcriptional HSR genes in pif4-1 or PIF4-OE were different and whether PIF4 could directly bind to other HS-inducible transcription factors (TFs) or HSP promoters. Does the overexpression of TaPIF4s improve the thermotolerance of wheat under severe HS? All of the above questions await further investigation in the future.

## 4. Materials and Methods

### 4.1. Plant Materials, Growth Conditions, and Heat Treatments

All of the plants used in this study were of Columbia-0 background. The *pif4-1* (SALK_140393C), *pifq,* and *PIF4-OE* used in this research were described previously [57,79,80]. The *35s:PIF4-TAP* (*His/Myc/Flag*) transgenic line was provided by Dr. Hongtao Liu [81]. All of the *Arabidopsis* seeds were sterilized and then incubated at 4 °C for 3 days in the dark. Next, seeds were sowed on ½ Murashige and Skoog (MS) medium containing 0.8% agar and 1% sucrose (pH 5.7).

For the thermotolerance assays, the seedlings were grown in darkness for 4 days and then treated at 45 °C for 1 h and recovered at 22 °C for 3 days under long day conditions (16 h light/8 h darkness). For the photomorphogenesis assays, the seedlings were grown under continuous red light (15 μmol m^−2^ s^−1^) for 4 days.

### 4.2. RNA-Seq Analysis

WT (Col-0) *Arabidopsis* were grown in normal conditions (dark or long day (16 h light/8 h darkness)) for 6 days, and AK-58 wheat cultivar seedlings were grown under normal conditions for 2 weeks and then treated with HS (37 °C), cold (5 °C), ABA (20 μM), Mannitol (200 mM), and NaCl (150 mM) before sampling. Each treatment was performed with two biological replicates. Samples were sequenced on the Illumina platform by Biomarker Technologies (Beijing, China). The expression levels were calculated using the FPKM method [82]. The FPKM values were normalized by log2 or log10 for heat maps. The heat maps were generated using TBtools [83].

To analyze the expression patterns of the *PIFs* in different tissues of *Arabidopsis* and *Triticum*, the transcriptomic data of *Arabidopsis* and Chinese Spring were downloaded from the *Arabidopsis* eFP Browser (http://bar.utoronto.ca/efp/cgi-bin/efpWeb.cgi, accessed on 11 April 2022) [84] and ExpVIP (http://www.wheat-expression.com/, accessed on 11 April 2022), respectively. The expression levels of *Arabidopsis* and Chinese Spring were calculated with the RPKM and TPM methods, respectively. The RPKM and TPM values were normalized by log2 for the heat maps. The heat maps were generated using TBtools [83].

### 4.3. Protein Extraction and Immunoblot Assays

The total proteins were extracted with lysis buffer (50 mM Tris-HCl pH 7.5, 150 mM NaCl, 1 mM EDTA, 10% (*vol*/*vol*) glycerol, 0.1%NP-40, 1 mM PMSF, and 1× complete Protease Inhibitor Mixture (Roche)). Samples were centrifuged at 4 °C (12,000× *g* for 10 min). Then, the supernatants were collected into new centrifuge tubes. Each protein sample was denatured at 95 °C for 10 min and then separated on 10% (*w*/*v*) SDS-PAGE gels. The proteins were transferred to a polyvinylidene fluoride (PVDF) membrane (Millipore) that was the same size. Then, the membrane was blocked with 5% skimmed milk powder. The proteins were immunoblotted with anti-Myc primary antibody (M20002, Abmart, 1: 5000 (*v*/*v*)) and horseradish peroxidase-conjugated anti-mouse IgG secondary antibody (AB0102, Abways, 1: 10,000 [*v*/*v*]). The chemiluminescence signals were captured with a Li-Cor/Odyssey system. The protein band intensities were calculated with ImageJ (https://imagej.nih.gov/ij/, accessed on 11 April 2022). The relative intensities were calculated with Histone3 (CY6587, Abways, 1: 5000 (*v*/*v*)) control. Immunoblot experiments were repeated for three biological replicates, essentially with the same conclusions, and the representative result is shown in Figure 6.

### 4.4. RNA Extraction and Quantitative Real-Time PCR Analysis

The total RNA was extracted with TRNzol Universal Reagent (DP424, TIANGEN). The quality of the RNA samples was detected by agarose gel electrophoresis using Nanodrop one for concentration determination. The RNA was reverse transcribed into cDNA with the PrimeScript™II 1st Strand cDNA Synthesis Kit (Takara, Kusatsu City, Japan, 6210A). qRT-PCR was performed using 2×TSINGKE Master qPCR Mix (SYBR Green I) (TSINGKE, TSE201) with the LightCycler^®^ 480 System (Roche, Basel, Switzerland). A two-step qPCR amplification program was used as follows: 95 °C for 1 min followed by 40 cycles of 95 °C for 10 s and 60 °C for 30 s. The cycle threshold (CT) values were calculated using the 2^−∆∆CT^ method. Each assay was performed in three technical replicates, and the relative expression levels were normalized to Actin2. The primer sequences used for qRT-PCR are listed in Supplemental Table S1.

### 4.5. Effector–Reporter Assays

To detect whether the PIF4 protein impacts *HSFA2 transcription* depends on whether or not G-box motifs are present. We constructed two different reporters for the effector–reporter assays. The *P_HSFA2_:LUC* reporter contained the *HSFA2* promoter driving LUC. The *P_HSFA2mGbox_:LUC* reporter indicated both the G-box mutations of *P_HSFA2_:LUC*. *35s:GFP* (negative control) and *35s:PIF4* were used as effectors. These constructs were transformed into *Agrobacterium tumefaciens* (strain GV3101). The GV3101 cultures harboring different reporters were cultured in liquid LB medium (50 mg/mL kanamycin and rifampicin) to an OD600_nm_ between 0.5 and 0.6. Then, *Agrobacterium* were collected after centrifugation (4000× *g* at 25 °C for 10 min) and resuspended using the infiltration buffer (10 mM MES pH 5.6, 150 μM Acetosyringone, and 10 mM MgCl_2_) to a final OD600_nm_ between 0.8 and 1.0. Then, *Agrobacterium* harboring *35s:GFP* or *35s:PIF4* were mixed in a 1:1 ratio and then infiltrated into *Nicotiana* leaves. The infiltrated *Nicotiana* was grown under normal conditions for 2 days. The *Nicotiana* leaves were infiltrated with 1mM D-fluorescein potassium salt before the luciferase activity was detected. LUC signaling was captured with the NightShade LB985 Plant Imaging System. The luciferase activity for each group was calculated using IndiGO software. All of the *35s:GFP* controls were set at 100%. The ratio represents the relative luminescence intensity of the *35s:PIF4* effector to the *35s:GFP* effector in the same reporter. Each assay was performed in at least four independent biological replicates, and representative results are shown.

### 4.6. ChIP-qPCR Assays

ChIP assays have been described in previous studies [85]. In brief, 7-day-old WT and *35s:PIF4-TAP* seedlings were crosslinked with 1% formaldehyde under vacuum. After 10 min, crosslinking was stopped by adding glycine to a final concentration of 125 mM. The seedlings were rinsed with water five times, and excessive moisture was removed with absorbent paper. The tissues were ground with 10 mL extraction buffer I (10 mM Tris-HCl pH 8, 0.4 M sucrose, 10 mM MgCl_2_, 5 mM BME, 1×protease inhibitor (Beyotime, Jiangsu, China, P1015), and 0.1 mM PMSF). The homogenate was filtered through a strainer (300 mesh) into a 15 mL falcon tube. Then, the filtered solution was centrifuged at 4000 rpm at 4 °C for 20 min. The supernatant was removed, and the pellet was resuspended in 1 mL extraction buffer II (10 mM Tris-HCl pH 8, 0.25 M sucrose, 1% Triton X-100, 10 mM MgCl_2_, 5 mM BME, 1mM EDTA, 1×protease inhibitor, and 0.1 mM PMSF). After centrifugation (12,000× *g* at 4 °C for 10 min), the supernatant was removed, and the pellet was resuspended in 300 μL of extraction buffer III (10 mM Tris-HCl pH 8, 1.7 M sucrose, 0.15% Triton X-100, 2 mM MgCl_2_, 5 mM BME, 1 mM EDTA, 1×protease inhibitor, and 0.1 mM PMSF). The solution was then laid on the top of a clean 300 μL of extraction buffer 3 and then centrifuged at 16,000× *g* at 4 °C for 1 h. The supernatant was removed, the chromatin pellet was resuspended in 200 μL nuclei lysis buffer (50 mM Tris-HCl pH 8, 1% SDS, 10 mM EDTA, 1×protease inhibitor, and 0.1 mM PMSF), and the tissues were then sonicated at 4 °C to 250~500 bp genomic DNA fragments. After centrifugation (14,000 rpm at 4 °C for 5 min), the supernatant was removed to a new tube, and 1.8 mL of ChIP dilution buffer (16.7 mM Tris-HCl pH 8, 1.1% Triton X-100, 1.2 mM EDTA, 167 mM NaCl, 1×protease inhibitor, and 0.1 mM PMSF) was added to dilute the 1% SDS to 0.1% SDS. The protein–DNA complex was immunoprecipitated by anti-Myc magnetic beads (Beyotime, P2118) and incubated while rotating at 4 °C overnight. The magnetic beads were attached to a magnet, and washed with 1 mL of low-salt wash buffer (20 mM Tris-HCl pH 8, 150 mM NaCl, 0.1% SDS, 1% TritonX-100, and 2 mM EDTA) two times, washed with high-salt wash buffer (20 mM Tris-HCl pH 8, 500 mM NaCl, 0.1% SDS, 1% TritonX-100, and 2 mM EDTA) two times, washed with LiCl wash buffer (10 mM Tris-HCl pH 8, 0.25 M LiCl, 1% NP40, 1% sodium deoxycholate, and 1 mM EDTA) two times, and, finally, washed with TE buffer (10 mM Tris-HCl pH 8, 1 mM EDTA) one time; each washing was performed at 4 °C for 5 min. After washing, the immunocomplexes were eluted from the magnetic beads twice with 250 μL of elution buffer (0.1 M NaHCO_3_ and 1% SDS); then, 20 μL 5 M NaCl was added to the eluate and reverse crosslinked at 65 °C for at least 6 h or overnight. Then, 10 μL of 0.5 M EDTA, 20 μL of 1 M Tris-HCl pH 6.5, and 2 μL of 10 mg/mL proteinase K were added to the eluate, and it was incubated for 1 h at 45 °C. The DNA was extracted using an equal volume of phenol/chloroform. The purified DNA was analyzed by qRT-PCR, which is described in Materials and Methods Section 4.4. The ChIP-qPCR values were normalized to TUB2. Additionally, the TA3 promoter was used as a negative control. Four technical replicates were performed for each ChIP-qPCR experiment. The primer sequences used for ChIP-qPCR are listed in Supplemental Table S2.

### 4.7. Homology Analysis of PIFs in Arabidopsis and Triticum

To further clarify the homology relationship of the PIFs in Arabidopsis and Triticum, the PIF protein sequences of Arabidopsis and Triticum were downloaded and compared. To download the sequences, we performed a BLASTP search using Ensemble Plants (http://plants.ensembl.org/index.html, accessed on 11 April 2022) and chose the sequences with e-values less than 1 × 10^−10^. Full-length protein alignments were performed by using MUSCLE [86], and phylogenetic trees were constructed using MEGA-X (https://www.megasoftware.net/, accessed on 11 April 2022). A total of 1000 bootstrap replications were performed using the neighbor-joining method. The tree was next modified by iTOL (https://itol.embl.de/, accessed on 11 April 2022). The MUF1 and bHLH domain sequences were defined using MEME (https://meme-suite.org/meme/tools/meme-chip, accessed on 11 April 2022).

### 4.8. Statistical Analysis

Statistical differences were calculated by Student’s *t*-test or one-way ANOVA. Asterisks indicate the significant differences (* *p* < 0.05). Different letters above each bar indicate statistically significant differences determined by Tukey’s multiple testing methods (*p* < 0.05).

## 5. Conclusions

In summary, we propose a hypothetical model that elucidates the molecular function of PIF4 in response to severe heat stress. PIF4 can directly bind to the G-box motifs of the HSFA2 promoter to activate its gene expression, thereby resulting in the activation of other HS-inducible genes, such as heat shock proteins. These lead to a stronger basal thermotolerance under non-heat-treatment conditions, thereby resulting in enhanced tolerance to severe heat stress. Meanwhile, the ATPIF4 homolog genes in wheat exhibited similar transcription patterns in response to heat stress. Our findings establish a molecular mechanism of PIF4 in mediating the heat stress response and provide a candidate direction for breeding heat-resistant wheat cultivars.

## Supplementary Materials

The following are available online at https://www.mdpi.com/article/10.3390/ijms23116017/s1.
