# Supplementary material for: PIF4 Promotes Expression of HSFA2 to Enhance Basal Thermotolerance in Arabidopsis
Source: Int J Mol Sci. 2022 May 27;23(11):6017. doi: 10.3390/ijms23116017 (PMC9181434; doi:10.3390/ijms23116017)
Supplement: Supplementary file 1 [file ijms-23-06017-s001.zip › Supplemental Figures.pdf]

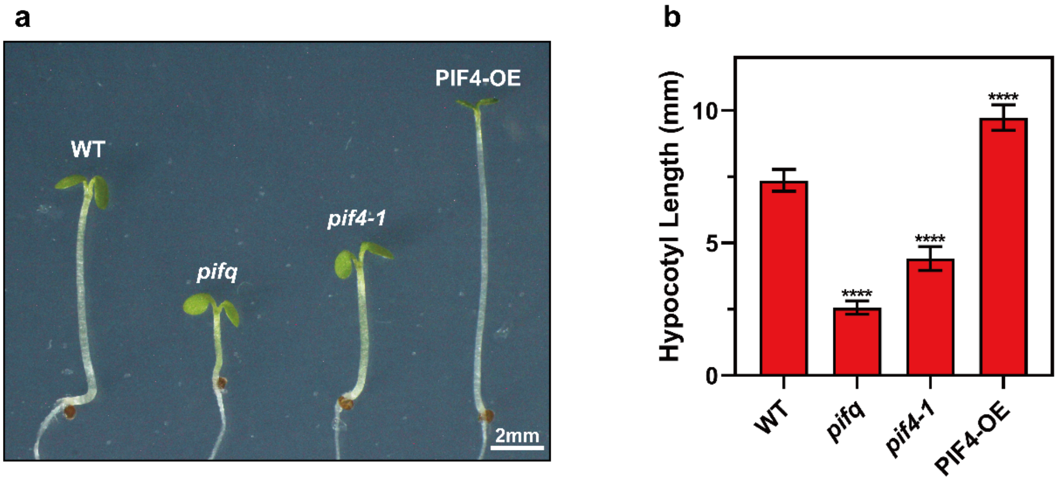

Figure S1. PIF4 is a positive regulator of hypocotyl elongation in photomorphogenesis.

(a) Phenotypes of 4-d–old Col-0, *pifq*, *pif4-1* and PIF4-OE seedlings grown under red light (15 μmol m^{-2} s^{-1}). Bar = 2 mm.

(b) Quantification of the hypocotyl length as indicated in (a). Values are means ± SD of at least 30 independent seedlings. Statistical differences were calculated by Student's *t*-test are shown. Asterisks indicate the significant difference (****P < 0.0001).

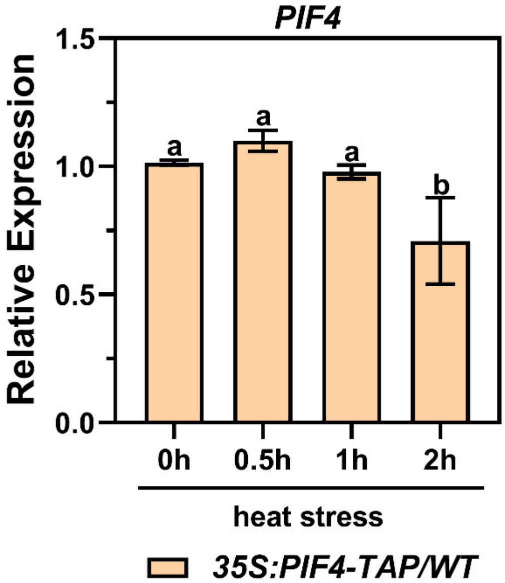

Figure S2. The transcription levels of *PIF4* were not induced by heat stress in *35S:PIF4-TAP/WT* seedlings.

qRT-PCR analysis of the expression level of *PIF4* in 4-d-old seedlings of *35S:PIF4-TAP/WT* grown in darkness and the seedlings transferred to 45°C for 0.5, 1, and 2 h. Error bars indicate mean ± SD. Actin2 was used as a reference gene. Statistical differences were calculated by one-way ANOVA. Different letters above each bar indicate statistically significant differences as determined by Tukey's multiple testing methods (P < 0.05).

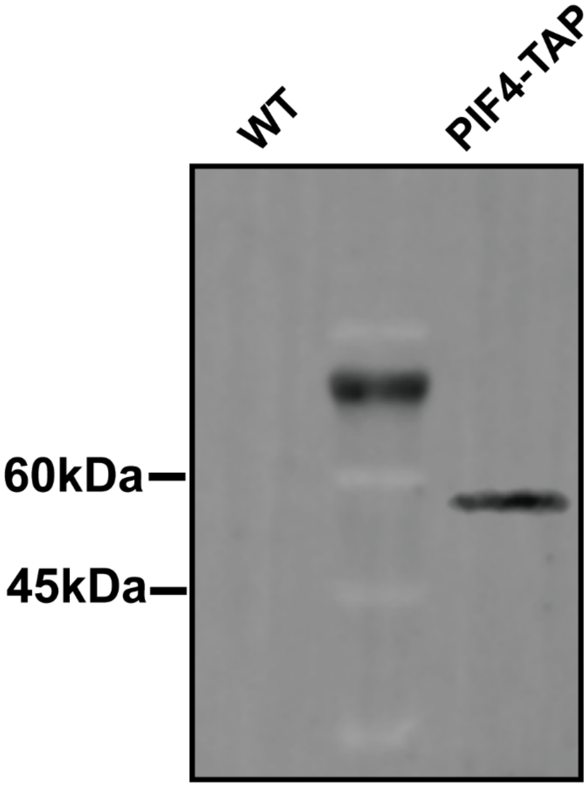

Figure S3. Detection of the PIF4-TAP protein in 35S:PIF4-TAP/WT and WT.

Detection of the PIF4-TAP protein in 4-d–old 35S:PIF4-TAP/WT and WT seedlings grown in darkness. The proteins were detected by immunoblot analysis using an anti-Myc antibody.
