# Supplementary material for: PIF4 Promotes Expression of HSFA2 to Enhance Basal Thermotolerance in Arabidopsis
Source: Int J Mol Sci. 2022 May 27;23(11):6017. doi: 10.3390/ijms23116017 (PMC9181434; doi:10.3390/ijms23116017)
Supplement: Supplementary file 1 [file ijms-23-06017-s001.zip › Supplemental Tables.pdf]

Table S1. Primer sequences for qRT-PCR analysis.

Accession	Gene	Primer sequence
AT3G18780	Actin2	F: GCACCCTGTTCTTCTTACCG
		R: AACCCTCGTAGATTGGCACA
AT2G26150	HSFA2	F: TTTGTTCAGCAGTTTGCGGTTAT
		R: TCCGTTTCCTCCCCACATC
AT3G51910	HSFA7a	F: AGAGGATCATCAACTGGTGGTAGAG
		R: AGCCAAACTCTCATCACTAAGCAAC
AT4G36990	HSFB1	F: GTTCGCCTTCTGAGTCTAATTCTGG
		R: CACGTTTAAGCTTCTCGTTCTCTCC
AT5G03720	HSP18.1	F: TTACCGGAGAATGCAAAGATG
		R: CGGAGATATCGATGGACTTGA
AT4G10250	HSP22.0	F: ACCGGTTCCCTGATCCATTCAAG
		R: GCCCTTCTGCTGTTTCTTTCCAGTC
AT3G12580	HSP70	F: GTACAAGGCTGAGGATGAAGAACAC
		R: TTAGATGCGATCTTCTCGTCCTTGA
AT4G24190	HSP90	F: GATGAACCGAACACTTTTGCTG
		R: GCATATCACCATCTTCCTCAACG
AT1G74310	HSP101	F: GCTTCAAATGAAAGACGTTGCTGTC
		TCCTTATAGGCCTAGCACCATACAC

Table S2. Primer sequences for ChIP-qPCR analysis.

Accession	Gene	Primer name	Primer sequence
AT5G62690	TUB2	TUB2-ChIP-F	ACAAACACAGAGAGGAGTGAGCA
		TUB2-ChIP-R	ACGCATCTTCGGTTGGATGAGTGA
AT1G37110	TA3	TA3 retrotransposon-ChIP-F	CTGCGTGGAAGTCTGTCAAA
		TA3 retrotransposon-ChIP-R	CTATGCCACAGGGCAGTTTT
AT2G26150	HSFA2	HSFA2-ChIP-P1-F	GTTGTGTTTGGGGAAGAACCGT
		HSFA2-ChIP-P1-R	ATATCTCGTGCGTGTCTCCA
		HSFA2-ChIP-P2-F	CTGGAACATTGTCTTGTCTTGTCG
		HSFA2-ChIP-P2-R	ATTGAGGCGTGGAGGAGAGAG
